# Optimization of extraction of genomic DNA from archived dried blood spot (DBS): potential application in epidemiological research & bio banking

**DOI:** 10.12688/gatesopenres.12855.2

**Published:** 2019-11-14

**Authors:** Abhinendra Kumar, Sharayu Mhatre, Sheela Godbole, Prabhat Jha, Rajesh Dikshit

**Affiliations:** 1 Centre for Cancer Epidemiology, Tata Memorial Centre, Mumbai, Maharashtra, 410210, India; 2 Homi Bhabha National Institute, Training School Complex, Anushaktinagar, Mumbai, 400094, India; 3 Department of Biostatistics and Epidemiology, National AIDS Research Institute, Pune, Maharashtra, 411026, India; 4 Li Ka Shing Knowledge Institiute, St Michael's Hospital, Center for Global Health Research, Toronto, ON, Canada

**Keywords:** Dried blood spot (DBS), Whatman 903 cards, FTA cards, Human genomic DNA, Bio-banking, Epidemiology

## Abstract

**Background: **Limited infrastructure is available to collect, store and transport venous blood in field epidemiological studies. Dried blood spot (DBS) is a robust potential alternative sample source for epidemiological studies & bio banking. A stable source of genomic DNA (gDNA) is required for long term storage in bio bank for its downstream applications. Our objective is to optimize the methods of gDNA extraction from stored DBS and with the aim of revealing its utility in large scale epidemiological studies.

**Methods: **The purpose of this study was to extract the maximum amount of gDNA from DBS on Whatman 903 protein saver card. gDNA was extracted through column  (Qiagen) & magnetic bead based (Invitrogen) methods. Quantification of extracted gDNA was performed with a spectrophotometer, fluorometer, and integrity analyzed by agarose gel electrophoresis.

**Result: **Large variation was observed in quantity & purity (260/280 ratio, 1.8-2.9) of the extracted gDNA. The intact gDNA bands on the electrophoresis gel reflect the robustness of DBS for gDNA even after prolonged storage time. The extracted gDNA amount 2.16 – 24 ng/µl is sufficient for its PCR based downstream application, but unfortunately it can’t be used for whole genome sequencing or genotyping from extracted gDNA. Sequencing or genotyping can be achieved by after increasing template copy number through whole genome amplification of extracted gDNA. The obtained results create a base for future research to develop high-throughput research and extraction methods from blood samples.

**Conclusion: **The above results reveal, DBS can be utilized as a potential and robust sample source for bio banking in field epidemiological studies.

## Introduction

The concept of using dried blood spot (DBS) in new born screening was presented by Guthrie and Susie in 1963 ^1^. DBS has been used for the last 5 decades by researcher in medical research. In field epidemiological studies there is a need for robust sample sources so they can be stored for long periods without any damage or spoilage. DBS is a much better option as compared to venous blood in low resource field setups for large epidemiological studies. Biomarkers reveals biological information from normal to disease condition and provide information about the disease condition, as it also acts as a prognostic marker. The collection of DBS is simple compared to venous blood collection as it only requires a finger prick, compared to venous puncture via needle for venous blood collection. Today DBS samples are utilized to test for a variety of health related markers including; infectious pathogens, HbA1c, total cholesterol, creatinine, uric acid, low density lipoprotein (LDL), high density lipoprotein (HDL), very low density lipoprotein (VLDL), Triglyceride and many more ^2– 4^. DBS is prepared by spotting 40–50µl of whole blood on Whatman 903 protein saver cards, air dried for 2 hours by hanging or by placing in a rack, and then packed it in sealed ziplock bags with desiccant. Other cards available for blood sampling include Ahlstrom, Whatman No.6, DMPK, FTA etc. however the Whatman 903 card is US Food and Drug Administration (FDA) approved for medical research ^5^. In epidemiological research the protein saver cards act as information storage devices in terms of blood based analytes and provide genetic, environmental, immunological information. Genomic DNA (gDNA) is a very robust and stable biological sample when stored on paper cards, and has been used for many decades ^3, 4^. RNA, which is less stable than gDNA in solution, appears to also be stable on DBS ^6^. Due to the small amount of blood in DBS, the obtained concentration of genetic material is also low but this problem can be overcome by amplification of the whole genome, and yield high quality DNA for performing assays, such as sequencing and genotyping arrays, at low cost ^7^. Limited studies are available regarding the use of DBS for downstream SNP genotyping following whole genome amplification ^6, 8^.

This study was performed with the aim of extracting maximal gDNA using archived DBS cards obtained from the Centre for Global Health Research (CGHR) Bangalore unit to establish its feasibility for downstream applications and biobanking in large scale epidemiological studies.

## Methods

### Ethical considerations and consent

The study was ethically approved by Institutional Ethics Review Board (IERB) of St. John’s Medical College and Hospital, Bangalore (India) with approval number IERB/1/77/05. After explaining the study to participants, informed written consent (as per norms of Indian Council of Medical Research (ICMR) Government of India) was obtained from volunteer participants.

### Subjects enrollment

This was part of a multicenter study involving the Centre for Global Health Research (CGHR) Bangalore unit and Tata Memorial Centre, Mumbai unit, who worked together to conduct study DBS. DBS samples were collected at health checkup camps in rural and urban areas of Bangalore city through a sample registration system (SRS). 3000 DBS samples were prepared during health checkup at Bangalore Centre. DBS samples were collected between years 2005–2007 & stored at 4°C, but later on it transported to Mumbai at ambient temperature in year 2013 while laboratory experiments were conducted in year 2016. DBS samples were prepared through finger prick method by using lancet (Accu Chek Softclix Lancet, Roche), puncture the finger site using lancet, drop of blood form which is lightly touch the circle of filter paper cards (GE Health Care Life Science, Catalog no. 10534612) and form valid DBS during health checkup camp by CGHR at Bangalore unit and transported to Tata memorial Centre (TMC) Mumbai for further analysis. Samples were collected between the years 2005–2007, but samples transported to Mumbai from Bangalore at ambient temperature in year 2013 and laboratory experiments conducted in 2016. DBS samples were collected by trained staff. Systematic random samples (n=40) were selected from top to bottom order from collected DBS. The following anthropometric measurements were recorded; height, weight, waist-to-hip ratio, blood pressure with gender and age. The complete study was explained to the subjects, and only voluntary participants aged between 18–49 years were included in this study after obtaining written informed consent as per the norms of Indian Council of Medical Research (ICMR) Government of India.

### Sample collection, transport and storage

**Figure 1.**
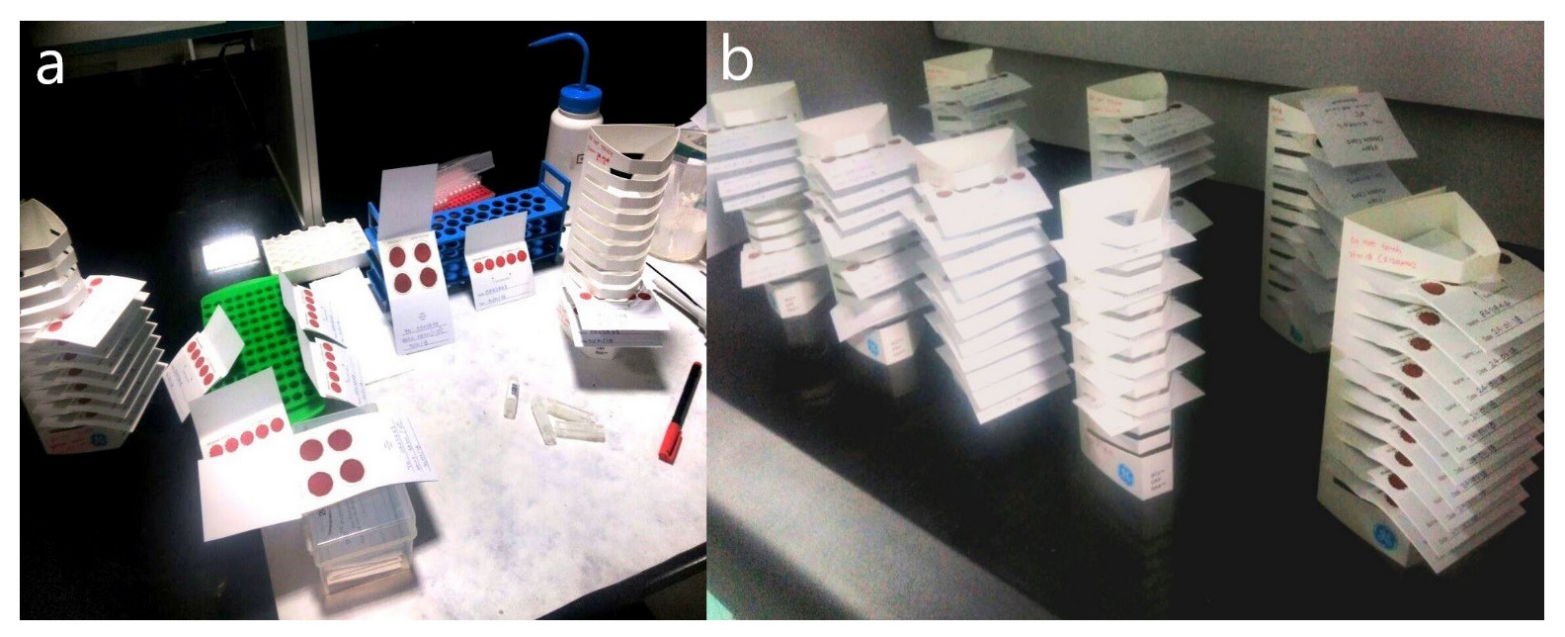
Preparation and drying of dried blood spot (DBS) cards. ( **a**) Prepared DBS cards. ( **b**) Drying of blood spots at room temperature.

***Finger prick blood DBS Preparation*.** Finger prick blood was collected using a lancet to puncture the fingertip. Once a full small drop of blood is formed it was lightly touched to the center of the circle on the filter paper (GE Health Care Life Science, Catalog no. 10534612) to form valid DBS. These were collected from study participants during health checkup organized at government schools, and at the center of villages. Cards were dried for 2 hours in velcro rack and packed in sealed ziplock bag with 1–2gm desiccant sachet ( Figure 1). Only valid DBS samples were used for gDNA extraction determined by the blood sample completely saturating each circle on the card, and not overlapping or merging with other blood circles. Prepared DBS samples were transported to the laboratory at the Centre for Cancer Epidemiology, Tata Memorial Centre, where they were stored in a -80°C refrigerator.

### Sample quality & validity

**Figure 2.**
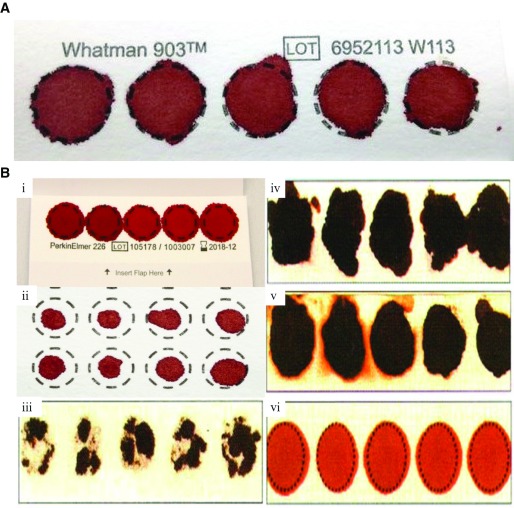
Valid / Invalid dried blood spot (DBS) specimens. ( **A**) **Valid specimen**. DBS with complete filled circle with proper air dry with no hemolyzed blood or serum ring. ( **B**) **Invalid DBS Specimen**. ( **i**) DBS with overlapping of spotted blood. ( **ii**) DBS with insufficient filled blood. ( **iii**) DBS with incomplete absorption which reduces blood volume. ( **iv**) DBS that is potentially rubbed and develop scratches. ( **v**) DBS with hemolyzed or contaminated blood. ( **vi**) DBS with improper air drying before packaging in ziplock bags.

We have used only good quality DBS samples for gDNA extraction, it is defined as the complete saturation of whole blood over the complete circle of blood collection card ^9^, blood card should be labelled and blood should adsorb on both side of the card. We have used only valid samples for gDNA extraction ( Figure 2).

### DBS processing

A generic single hole 6mm punch plier was used to cut the blood spots from the Whatman 903 paper card. A punch of diameter 6mm represents approximately 8.7±1.9µl of blood spotted ^10^. 1 – 4 blood spots of size 6 mm punch was added to an eppendorf tube and incubated with 200µl PBS (readymade PBS buffer used with pH 7.4 supplied by Gibco with Ref No. 10010-023) overnight at room temperature. Our major aim was to extract the maximum amount of gDNA, therefore we have used 6mm × 1 spot to 6mm × 4 spots for extracting gDNA from DBS ( Table 1). We have used phosphate buffer saline (PBS) for extraction of completely dry blood matrix on Whatman 903 for easy gDNA extraction, because once the surface of blood spot becomes wet, it is easy to extract DNA from the adsorbed blood.

**Table 1.** Average genomic DNA (gDNA) concentration with different number of blood spots.

Number of blood spots used of size 6mm	Average gDNA concentration (ng/µl)+SE ^M^	Average 260/280 ratio	Total elution volume	Total gDNA Yield
6mm × 1 spot (n=10)	3.43 ± 0.2893	1.93	30µl	102.9 ng
6mm × 2 spots (n=10)	6.38 ± 0.3540	2.18	30µl	191.4 ng
6mm × 3 spots (n=10)	7.23 ± 0.2491	2.30	30µl	216.9 ng
6mm × 4 spots (n=10)	8.91 ± 1.6863	2.84	30µl	267.3 ng

### Genomic DNA extraction methods

We have applied 2 methods for gDNA extraction from DBS. ( **1**) Column based (QIAamp DNA kit, Qiagen) ( **2**) Magnetic bead based (ChargeSwitch Forensic DNA Purification Kit, Invitrogen).

***Column based gDNA extraction from DBS*.** We used the QIAamp DNA kit (Qiagen, Catalog no. 56304). 1 – 4 blood spots 6mm in size were added with 180 µL of cell lysis buffer ATL (Lysis buffer supplied with Qiagen kit), and incubated in a waterbath (Trishul Equipment, Sr. No. 5460311) at 85°C for 10 min. 20 µL Proteinase K was added and incubated it at 56°C for 1 hour to denature the proteins. 3–4 µL RNAse was added immediately after to degrade RNA, then 200 µL buffer AL (Lysis buffer supplied with Qiagen kit) was added & mixed thoroughly by vortexing and incubated at 70°C in a waterbath (Trishul Equipment with Sr. No. 5460311) for 10 min. Buffer AL helps in complete cell lysis and binding of gDNA with the silica gel of the column provided in the Qiagen kit. gDNA was then immediately precipitated by adding 200 µL of 70% v/v ethanol. The solution was then transferred into a spin column (supplied with Qiagen kit) and centrifuged (Eppendorf 5810R) at 8000 rpm for 1 min. The spin column has the capacity to load approximately 600 microliter sample at a time, but generally we had approximately 1.2 or 1.4 ml of solution, we therefore performed the process 2–3 times. In this process gDNA becomes bound with the column, impurities are then washed out with 700 µL buffer AW1 (Wash buffer with a low concentration of quinidine) followed by 700 µL of AW2 (Wash buffer with Tris based ethanol solution used for removal of salts) buffer. Buffers AW1 & AW2 remove unwanted impurities from the gDNA. The empty tube was then centrifuged at 14000 rpm for 3 min for complete removal of ethanol from the gDNA. Finally the gDNA was eluted with 30 µl of pre-incubated elution buffer (AE).

***Magnetic bead based gDNA extraction from DBS*.** We used the ChargeSwitch Forensic DNA Purification Kit (Thermofisher, Catalog No. CS11200). Processed DBS samples were added to 1ml lysis buffer with 10 µL of Proteinase K in a tube, vortexed, and incubated at 55°C in a waterbath (Trishul Equipment Sr. No. 5460311) for 1 hour. Blood spots were removed after complete cell lysis and 200 µL of purification buffer added, followed by 20 µL magnetic beads. The solution was then gently mixed, left for 5 minutes, and then incubated on a Magna Rack (Thermofisher, catalog no. AM10027) for 1 minute. The supernatant was removed after complete binding of the pellet to the magnet of the Magna Rack. The pellet containing gDNA was then washed with 500 µL wash buffer (W12) 3 times and finally DNA eluted with 30–60 µL Elution Buffer (E5). We have followed the protocol as per manufacturer recommendation with some modifications; incubation time was increased from 4 hours to overnight for complete extraction of eluate from filter paper, and we also increased the time for proper binding of gDNA with the spin column.

### Genomic DNA quantification

Concentration of extracted gDNA was measured using a Qubit 3.0 fluorometer and purity was measured by a spectrophotometer. Quality and integrity of gDNA was checked by performing a 0.8% Agarose gel electrophoresis.

Qubit 3.0 Fluorometer (Thermofisher, Catalog No. Q32850) was used to measure gDNA concentration by taking 1 µl gDNA sample in 0.2ml PCR tube with 200 µl buffer (199µl dsDNA BR buffer + 1 µl Ethidium Bromide dye) after proper mixing for 1min. Concentration was then measured with a fluorometer.

A spectrophotometer at wavelength 260/280 ratio (NanoDrop 2000 Thermofisher) was used to check purity of gDNA, by applying 1 µl gDNA sample directly to the device and measuring the 260/280 ratio.

Agarose gel electrophoresis was performed following preparation of a 0.8% agarose gel which was loaded with 5 µl gDNA in wells and run at 70–80 volts for 2 hours. A gel photograph was captured using a gel dock (Model: UVP EC3-Imaging System).

### Statistical analysis

All the data analysis done by using excel to calculate average, total yield and standard error of mean (SE _M_: SD/√N), where SD is standard deviation, N is total number of samplese.

## Results

### Genomic DNA extraction efficiency

**Figure 3.**
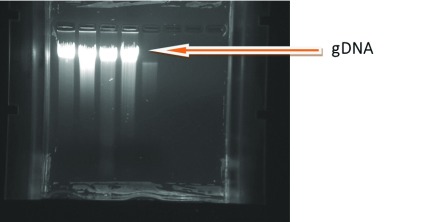
Agarose gel electrophoresis of extracted gDNA from dried blood spots. Figure shows highly intense bands which are mostly intact with little smear. 5μl DNA loaded in lane1 & lane3 with concentration 7.19 ng/μl & 5μl DNA loaded in lane2 & lane 4 with concentration 8.7 ng/μl.

Table 1 showed, average amount of gDNA extracted from DBS with their average yield. In our findings, we observed large variation in the concentration, from 2.16ng/µl to 24ng/µl, of the extracted gDNA ( Table 2). The integrity of gDNA was checked using 0.8% agarose gel electrophoresis, and highly intense single bands were observed on the gel ( Figure 3). The purity of gDNA was measured at an absorbance of 260/280nm (1.8–2.0) ratio. If it is less than 1.8 or greater than 2.0 it indicates the presence of impurities in the genetic material. gDNA concentration of each DBS with different number of blood spots, with their purity and total gDNA yield are presented in Table 3.

**Table 2.** Genomic DNA quantification (gDNA) by Fluorometer on stored dried blood spot (DBS) at standard condition.

Type of filter paper	gDNA concentration range (ng/µl)	Elution volume	Total Yield (ng)
Whatman 903 card	2.16 ng/µl - 24 ng/µl	30 µl	64.8 ng – 720 ng

**Table 3.** Genomic DNA (gDNA) concentration of each dried blood spot (DBS) from different number of blood spots.

Total number of blood spots used	DNA Yield (ng/µl)	260/280 ratio	Total Elution Volume	Total Yield
6mm × 1 spot	3.4	1.84	30	102
6mm × 1 spot	3.52	1.9	30	105.6
6mm × 1 spot	4.52	1.8	30	135.6
6mm × 1 spot	4.7	1.39	30	141
6mm × 1 spot	2.16	2.4	30	64.8
6mm × 1 spot	2.19	1.98	30	65.7
6mm × 1 spot	2.12	2.1	30	63.6
6mm × 1 spot	4.66	2.23	30	139.8
6mm × 1 spot	3.56	2.21	30	106.8
6mm × 1 spot	3.47	1.5	30	104.1
6mm × 2 spots	4.98	2.4	30	149.4
6mm × 2 spots	5.95	2.2	30	178.5
6mm × 2 spots	6.36	2.23	30	190.8
6mm × 2 spots	6.4	2.13	30	192
6mm × 2 spots	8.98	2.28	30	269.4
6mm × 2 spots	8.06	2.32	30	241.8
6mm × 2 spots	5.4	2.1	30	162
6mm × 2 spots	5.49	1.82	30	164.7
6mm × 2 spots	6.4	2.15	30	192
6mm × 2 spots	5.82	2.21	30	174.6
6mm × 3 spots	6.86	2.45	30	205.8
6mm × 3 spots	8.08	2.61	30	242.4
6mm × 3 spots	5.72	2.28	30	171.6
6mm × 3 spots	7.13	2.41	30	213.9
6mm × 3 spots	6.94	2.21	30	208.2
6mm × 3 spots	7.18	2.51	30	215.4
6mm × 3 spots	8.7	1.98	30	261
6mm × 3 spots	6.43	2.31	30	192.9
6mm × 3 spots	8.12	2.38	30	243.6
6mm × 3 spots	7.19	1.95	30	215.7
6mm × 4 spots	9.9	2.9	30	297
6mm × 4 spots	10.9	2.84	30	327
6mm × 4 spots	7.6	2.93	30	228
6mm × 4 spots	8.23	2.74	30	246.9
6mm × 4 spots	9.25	2.62	30	277.5
6mm × 4 spots	24	2.89	30	720
6mm × 4 spots	3.36	2.84	30	100.8
6mm × 4 spots	7.62	2.91	30	228.6
6mm × 4 spots	4.17	2.86	30	125.1
6mm × 4 spots	4.12	2.96	30	123.6

This amount of gDNA extracted can be used for polymerase chain reaction (PCR), and PCR based molecular assays such as PCR based sequencing, PCR based genotyping, but can’t be used for whole genome sequencing or genotyping ^11^.

## Discussion

For the last 5 decades, DBS sample have been collected and stored in bio-banks to conduct field epidemiological studies worldwide. DBS collection on filter paper is more applicable and acceptable method in epidemiological research as compared with standard venous blood. The advantage of DBS over venous blood collection include less discomfort for the subject, especially if many samples are needed within a short period of time, only a small amount of blood is needed to perform the assay. Our findings shows that, we can extract the gDNA from dried blood spots. Previously, studies have been performed to compare genomic DNA extraction methods to examine its feasibility in genetic studies ^12^. As per obtained results, we have found good concentration of total gDNA, In this study, our target was to improve the maximum extraction of gDNA from DBS. We followed 2 methods; one column based and one magnetic bead based. Before proceeding to cell lysis process, we had treated the blood spots with PBS (pH 7.4) & kept it overnight at 37°C to elute the complete matrix from the Whatman for efficient & complete cell lysis. DNA samples can be stable on filter paper for many years if it is stored in dry conditions ^8^. Our main objective was to extract the maximum amount of gDNA from DBS irrespective of methodology use, therefore we have used 2 methods to extract gDNA to evaluate from which method we have got more gDNA but unfortunately, we have not found any difference in gDNA concentration with between both methods. We did assay randomly from both methods with full focus on maximum quantity of gDNA extraction from DBS. We have tried gDNA extraction with direct cell lysis of DBS by using lysis buffer and also blood spots treated with PBS overnight to complete elution of eluate. We have done these experiments to evaluate gDNA concentration but unfortunately there are no such yield increases with these modifications. As our results show, there is large variation in the concentration and purity (260/280) of extracted gDNA in both the methods. This variation might be due to the small volume of blood, long term storage, loss during assays, cell debris, cellulosic component of the Whatman card etc. In the case of column (QIAamp) based gDNA extraction, 5% loss is predicted by the manufacturer, where as in magnetic bead based DNA extraction, some 5–10% beads are lost during assay, results there is loss of gDNA. The obtained purity also shows variation, 260/280 ratio 1.8–2.9, this might be due to interference of cellulosic components of Whatman paper. It is true that a 260/280 ratio >2.0 indicates impurities. But due to limitation of blood spots we have not increased the number of spots beyond 4. As I have mentioned that we have got DNA concentration in a range 64.8ng – 720ng. This amount of DNA can be used in downstream applications and we can remove the impurities by gel purification method.

gDNA concentration depends on the blood matrix on spots. In a study using DBS stored for 6 years and they found reduced gDNA concentration in quality and quantity. ^13^, but some other studies reported that gDNA is stable for at least 11 years under ambient tropical conditions ^8^. We have performed some modification to the protocol for the extraction, we increased the time for cell lysis, binding of DNA with column & beads, washing with buffer & final elution. Due to regular successive research on DBS, today DBS samples are used for genetic analysis, proteome research, vitamins estimation, infection agent, epigenetic research, nucleic acid research ^14– 17^.

A punch of diameter 6mm represents approximately 8.7±1.9µl of blood spotted. This difference in blood volume from a single spot might be due to the presence of hematocrit, because due to increased percentage of hematocrit in blood, the blood becomes very viscous and it can’t spread homogenously over the Whatman circle which results the concentration of DNA and blood on 6mm spot changes accordingly. Composition of hematocrit value influence the gDNA concentration with different number of blood spots. Filter paper contains cellulosic fibers, probably referred as cotton linters, while extracting gDNA from Whatman card, the cellulosic composition of filter paper interfere the concentration and purity of gDNA because during thermal agitation and vortex steps in protocol, these cotton linters are also present in supernatant and interfere with final DNA elution steps.

Our findings show, approximately 64.8ng – 720ng gDNA is extracted from Whatman 903 card >50 ng is sufficient for PCR based applications ^18^. DBS extracted gDNA can be used in downstream applications such as polymerase chain reaction (PCR), PCR based sequencing, PCR based genotyping, disease diagnosis, molecular basis of disease etiology and study of genetic variants. the extracted amount of gDNA, however, cannot be used for whole genome sequencing, but this can be overcome by whole genome amplification of extracted gDNA, as this will increase the concentration of gDNA by increasing the copy number of templates. DBS is a suitable and applicable sample source in large scale epidemiological studies and biobanking. Further study is warranted to explore DBS efficiency in high throughput research to reveal other biochemical analytes stability on filter paper card to replace venous blood collection in future epidemiological studies.

## Conclusion

Analyte stability on filter paper in dry form is a good biological sample source to perform molecular epidemiological based assays. Dried blood spot on paper card act as a potential and robust sample source for biobanking in large scale epidemiological studies.

## Data availability

Data underlying this study is available from Open Science Framework. Dataset 1: Optimization of extraction of gDNA from DBS: Potential application in epidemiological research & biobanking. http://doi.org/10.17605/OSF.IO/FZYTM ^19^

Data is available under a CC0 1.0 Universal license.
