# Supplementary material for: Optimization of extraction of genomic DNA from archived dried blood spot (DBS): potential application in epidemiological research & bio banking
Source: Gates Open Res. 2019 Nov 14;2:57. Originally published 2018 Nov 14. [Version 3] doi: 10.12688/gatesopenres.12855.2 (PMC6883222; doi:10.12688/gatesopenres.12855.2)
Supplement: Peer review history

# Peer review: Reviewer response for version 3

**DOI:** 10.21956/gatesopenres.14229.r28253

**Published:** 2019-12-05

**Authors:** Nicklas H. Staunstrup

**Affiliations:** 1 Department of Biomedicine, University of Aarhus, Aarhus, Denmark

Kumar and authors compare two gDNA extraction approaches from dried blood spots (DBSs) with the intent to optimize their usability in epidemiological studies. 

 I have the following comments:

- Description of biomarkers in the introduction is too simplistic. Biomarkers can both be of e.g. prognostic, diagnostic, or predictive value and they are not always associated with disease but e.g. exposure or any normal phenotype. Please revise.
- The statement that there is a limited knowledge concerning the use of DBSs for genotyping is not true. Several published works by the iPSYCH and PGC consortium proves otherwise. Please revise.
- In general references are often outdated, leading to false conclusions. The authors should revise their reference list.
- Describe the process of picking randomized samples in greater detail. Also, the wording “systematic random samples” is contradictive. Please revise.
- Why did the authors record meta-data (height etc.) on the participants? There appears to be no correction for any of these parameters.
- The paragraphs “subjects enrollment” and “sample transportation…” should be merged as essentially the same samples are being introduced twice, making it difficult to follow.
- There seems to be no actual comparison between the two extraction methods applied, which appears strange as this is the main aim? A t-test or ANOVA should be performed (depending on set-up) comparing the two methods.
- Where does the conc. range 64.8-720ng come from? How does it compare to table 1?
- How does the used methods and outcome compare to other used approaches, e.g. the one used by Hollegaard (ref 7 and 13)?
- Rephrase the last paragraph in results section. Sequencing e.g. MeDIP-seq and MBD-seq has been performed on unamplified DBS gDNA and genotyping (as the authors also write) can be done on amplified gDNA. So, both approaches are feasible.
- Removing impurities by gel purification is not an optimal approach as DNA is lost and impurities from the gel material will be present.
- The language in general requires improvement, there are many grammar and spelling mistakes.

I confirm that I have read this submission and believe that I have an appropriate level of expertise to confirm that it is of an acceptable scientific standard, however I have significant reservations, as outlined above.

# Peer review: Reviewer response for version 3

**DOI:** 10.21956/gatesopenres.14229.r28232

**Published:** 2019-11-28

**Authors:** Anubhav Tripathi

**Affiliations:** 1 Center for Biomedical Engineering, School of Engineering, Brown University, Providence, RI, USA

The revised version addresses all the issues raised by the reviewers.

I confirm that I have read this submission and believe that I have an appropriate level of expertise to confirm that it is of an acceptable scientific standard.

# Peer review: Reviewer response for version 3

**DOI:** 10.21956/gatesopenres.14229.r28231

**Published:** 2019-11-28

**Authors:** Lakshmy Ramakrishnan, Ransi Ann Abraham

**Affiliations:** 1 Department of Cardiac Biochemistry, All India Institute of Medical Sciences (AIIMS), New Delhi, India

Grammar still needs to be corrected in several places.

We confirm that we have read this submission and believe that we have an appropriate level of expertise to confirm that it is of an acceptable scientific standard.

# Peer review: Reviewer response for version 2

**DOI:** 10.21956/gatesopenres.14166.r27922

**Published:** 2019-10-15

**Authors:** Anubhav Tripathi

**Affiliations:** 1 Center for Biomedical Engineering, School of Engineering, Brown University, Providence, RI, USA

The paper “Optimization of extraction of genomic DNA from archived Dried Blood Spot (DBs): potential application in epidemiological research and bio-banking” is a well characterized study of gDNA extraction from epidemiological samples. However, the reviewer still has some significant concerns with the material presented as outlined below:

 Concerns:

- **Innovation:**
  - This paper deals specifically with gDNA extraction from DBS in biobanking and epidemiology. However, it remains unclear to the reviewer how this work significantly builds upon the substantive literature on DNA extraction and longevity in DBS that exists in the literature (some of which is nicely summarized within this work).
  - The stated gap in knowledge the authors are looking to address is “the use of DBS for downstream SNP genotyping following whole genome amplification”. How does this differ from previous works that have investigated DBS quality in relation to SNP genotyping (such as Rajatileka *et al.*, 2013 ^1^)?
  - The reasons for needing a separate optimized process are not made clear within the paper.
- **Methods:**
  - Please add a section outlining calculations and statistical methods.
  - How was total yield calculated?
  - How many blood spots were processed for each method? Replicates?
  - The addition of statistical methods (e.g. Standard error of the mean) would greatly aid readers in interpretation of datasets.
- **Figures:**
  - All figures and tables: The process of extraction used in each sample needs to be clearly represented on all figures and tables.
  - Table 2: The purpose of table 2 is unclear as this information is already displayed in both tables 1 and 3.
  - Figure 3:
  - It is unclear if these four samples are different blood spots, different testing methods or just replicates. Please label on gel image.
  - Lane 2 does look degraded – more so than the other lanes. Is there a reason for this degradation?
- **Minor comments:**
  - Table 3 — The units on the column marked total yield are missing.

I confirm that I have read this submission and believe that I have an appropriate level of expertise to confirm that it is of an acceptable scientific standard, however I have significant reservations, as outlined above.

## References

[1] : Isolation of human genomic DNA for genetic analysis from premature neonates: a comparison between newborn dried blood spots, whole blood and umbilical cord tissue. *BMC Genet*.2013;14: 10.1186/1471-2156-14-105 105 10.1186/1471-2156-14-105 24168095PMC3817355

# Peer review: Reviewer response for version 2

**DOI:** 10.21956/gatesopenres.14166.r27514

**Published:** 2019-09-26

**Authors:** Lakshmy Ramakrishnan, Ransi Ann Abraham

**Affiliations:** 1 Department of Cardiac Biochemistry, All India Institute of Medical Sciences (AIIMS), New Delhi, India

The authors have attempted to answer the queries raised by us. 

1. Since two methods of DNA extraction were employed we expected two sets of values in Table 3 (DNA yield both the methods separately).
2. It is not clear at what temperature the DBS samples were stored in Bangalore from 2006-2007 till 2013 when the samples were shifted to Mumbai. 
3. The grammar and language still needs correction in many places, for instance it is not clear what the authors want to convey in the statement "We obtained average gDNA concentrations of 6mm × 1 spot to 6mm × 4 spots from DBS cards" under the results section or the sentence "3000 DBS samples were prepared through finger prick method by using lancet (Accu Chek Softclix Lancet, Roche), puncture the finger site using lancet, drop of blood form which is lightly touch the circle of filter paper cards (GE Health Care Life Science, Catalog no. 10534612) and form valid DBS during health checkup camp by CGHR at Bangalore unit and transported to Tata memorial Centre (TMC) Mumbai for further analysis. Samples were collected between the years 2005–2007, but samples transported to Mumbai from Bangalore at ambient temperature in year 2013 and laboratory experiments conducted in 2016" under subject enrollment.

We confirm that we have read this submission and believe that we have an appropriate level of expertise to confirm that it is of an acceptable scientific standard, however we have significant reservations, as outlined above.

# Peer review: Reviewer response for version 1

**DOI:** 10.21956/gatesopenres.13936.r26761

**Published:** 2018-11-28

**Authors:** Lakshmy Ramakrishnan, Ransi Ann Abraham

**Affiliations:** 1 Department of Cardiac Biochemistry, All India Institute of Medical Sciences (AIIMS), New Delhi, India

Blood collected and stored as DBS is a very attractive and non-invasive alternative for measurement of several analytes. The only caveat is that the analyte of interest should be stable on drying and should be selectively eluted from blood with least interference from other contaminants.

 The paper by Kumar et al. describes the utility of DBS as a source of gDNA for PCR based molecular assay. The authors have extracted DNA by two methods: column based gDNA extraction and magnetic based gDNA extraction. It is not clear as to why two methods of DNA extraction was employed. The results do not show comparison of yield or quality of DNA by the two methods, Was one method superior to other? If all the 40 samples were extracted by both the methods the result should indicate the 260/280 ratio and yield for both the methods. It is also not clear if the DBS sample processed by overnight incubation in PBS was used as starting material for both the methods. In the column based method it is mentioned that spots were added to lysis buffer whereas in the magnetic bead based method processed DNA samples were added.

 A 260/280 ratio >2.0 is indicative of impurity as mentioned by the authors also. Most samples showed ratio >2.0 specially when the number of spots were increased. How is this likely to influence the downstream applications should be mentioned in the discussion. Since the authors have not demonstrated that the extracted DNA is suitable for downstream applications by actually performing some assays, this should be mentioned as limitation of the study.

 Several papers have been published on extraction of DNA from DBS, some authors have described increasing the yield by employing various means ^1^^-^^2^^-^^3^. A mention of these papers in the discussion would help the readers.

 In the method section the following information would be useful - since the spotted blood samples were left at room temperature for drying, the ambient temperature during collection period should be mentioned. It is not clear as to how long the samples were kept at Bangalore before transportation to Mumbai and at what temperature? At what temperature were the DBS samples when they were transported to Mumbai?

 The manuscript need to be edited to avoid repetitions of information and also language and grammar editing would improve the quality of the paper.

We confirm that we have read this submission and believe that we have an appropriate level of expertise to state that we do not consider it to be of an acceptable scientific standard, for reasons outlined above.

## References

[1] : Rapid DNA extraction from dried blood spots on filter paper: potential applications in biobanking. *Osong Public Health Res Perspect*.2014;5(6) : 10.1016/j.phrp.2014.09.005 351-7 10.1016/j.phrp.2014.09.005 25562044PMC4281615

[2] : Cost-effective and scalable DNA extraction method from dried blood spots. *Clin Chem*.2013;59(7) : 10.1373/clinchem.2012.198945 1045-51 10.1373/clinchem.2012.198945 23509109

[3] : Superparamagnetic-bead Based Method: An Effective DNA Extraction from Dried Blood Spots (DBS) for Diagnostic PCR. *J Clin Diagn Res*.2014;8(4) : 10.7860/JCDR/2014/8171.4226 FC01-4 10.7860/JCDR/2014/8171.4226 24959449PMC4064838

# Response

**Published:** 2019-06-17

**Authors:** Prabhat Jha

**Affiliations:** University of Toronto, Canada

**Our article 12855 – responses to comments by reviewers Dr. Lakshmy Ramakrishnan (AIIMS, New Delhi) and Dr. Ransi Ann Abraham (AIIMS, New Delhi)**

**Dr. Lakshmy and Dr. Ransi**: Blood collected and stored as DBS is a very attractive and non-invasive alternative for measurement of several analytes. The only caveat is that the analyte of interest should be stable on drying and should be selectively eluted from blood with least interference from other contaminants.

**Dr. Lakshmy and Dr. Ransi**: The paper by Kumar et al. describes the utility of DBS as a source of gDNA for PCR based molecular assay. The authors have extracted DNA by two methods: column based gDNA extraction and magnetic based gDNA extraction. It is not clear as to why two methods of DNA extraction was employed. The results do not show comparison of yield or quality of DNA by the two methods, Was one method superior to other? If all the 40 samples were extracted by both the methods the result should indicate the 260/280 ratio and yield for both the methods. It is also not clear if the DBS sample processed by overnight incubation in PBS was used as starting material for both the methods. In the column based method it is mentioned that spots were added to lysis buffer whereas in the magnetic bead based method processed DNA samples were added.

**Reply by Abhinendra**: Our main objective was to extract the maximum amount of gDNA from DBS irrespective of methodology use, therefore we have used 2 methods to extract gDNA to evaluate from which method we have got more gDNA but unfortunately we have not found any difference in gDNA concentration with between both methods. We did assay randomly from both methods with full focus on maximum quantity of gDNA extraction from DBS. We have tried gDNA extraction with direct cell lysis of DBS by using lysis buffer and also blood spots treated with PBS overnight to complete elution of eluate. We have done these experiments to evaluate gDNA concentration but unfortunately there are no such yield increases with these modifications.

**Dr. Lakshmy and Dr. Ransi**: A 260/280 ratio >2.0 is indicative of impurity as mentioned by the authors also. Most samples showed ratio >2.0 specially when the number of spots were increased. How is this likely to influence the downstream applications should be mentioned in the discussion. Since the authors have not demonstrated that the extracted DNA is suitable for downstream applications by actually performing some assays, this should be mentioned as limitation of the study.

**Reply by Abhinendra**: Yes it is true that a 260/280 ratio >2.0 indicates impurities. But due to limitation of blood spots we have not increased the number of spots beyond 4. As I have mentioned that we have got DNA concentration in a range 64.8ng – 720ng. This amount of DNA can be used in downstream applications and we can remove the impurities by gel purification method.

**Dr. Lakshmy and Dr. Ransi**: Several papers have been published on extraction of DNA from DBS, some authors have described increasing the yield by employing various means ^1^ ^- 2- 3^. A mention of these papers in the discussion would help the readers. In the method section the following information would be useful - since the spotted blood samples were left at room temperature for drying, the ambient temperature during collection period should be mentioned. It is not clear as to how long the samples were kept at Bangalore before transportation to Mumbai and at what temperature? At what temperature were the DBS samples when they were transported to Mumbai?

The manuscript need to be edited to avoid repetitions of information and also language and grammar editing would improve the quality of the paper.

**Reply by Abhinendra**: DBS samples were transferred from Bangalore to Mumbai at ambient temperature.
